# The Effects of Risperidone on Cognition in People With Autism Spectrum Disorder: A Systematic Review

**DOI:** 10.7759/cureus.45524

**Published:** 2023-09-19

**Authors:** Jhenelle Hutchinson, Oluwafolawemi Adefokun, Parikshit Bittla, Simran Kaur, Vani Sojitra, Anam Zahra, Safeera Khan

**Affiliations:** 1 Psychiatry, California Institute of Behavioral Neurosciences & Psychology, Fairfield, USA; 2 Medicine, California Institute of Behavioral Neurosciences & Psychology, Fairfield, USA; 3 Internal Medicine, California Institute of Behavioral Neurosciences & Psychology, Fairfield, USA; 4 Medicine, Bavadia Hospital, Una, Gujarat, IND; 5 Surgery, California Institute of Behavioral Neurosciences & Psychology, Fairfield, USA

**Keywords:** risperidone, risperidone therapy, risperidone adverse effect, risperidone toxicity, cognitive function, cognition, autism spectrum disorder (asd)

## Abstract

Autism spectrum disorder is made up of several disorders, which include autism, Asperger syndrome, and pervasive developmental disorder. Boys are four times more likely to be diagnosed than girls with autism spectrum disorder, and symptoms usually become apparent by the age of three. Autism spectrum disorders’ core characteristic features are abnormal interaction, impairment in communication, and stereotyped behaviors with restricted activities and interests. There are also non-core features associated with autism spectrum disorder, and these are aggression, self-injurious behavior, and tantrums. To date, there is no one drug approved to treat the core symptoms of autism spectrum disorder, but antipsychotic drugs such as risperidone have been shown to be effective at treating both core and non-core symptoms in controlled trials using multiple behavioral rating scales such as the Aberrant Behavioral Checklist subscale, the Clinical Global Impression Improvement Scale, the Ritvo-Freeman Real Life Scale, the Children’s Yale-Brown Obsessive Compulsive Scale, the Vineland Adaptive Behavior Scale, and the Social Withdrawal Subscale. The safety, efficacy, acceptability, and tolerability of risperidone were assessed in these studies, and weight gain was a common side effect observed, but the outcome was usually mild and self-limiting. The effect of risperidone on cognition was explored in this article. The studies selected for this article were of small sample size and short duration, which presented limitations for treatment with risperidone and an area that needs to be explored further for its contribution to clinical practice.

## Introduction and background

Autism spectrum disorder was initially considered to be a rare disease. However, recently, the prevalence reported has substantially increased. Most recently, it has been estimated that the prevalence is one in 68 children at eight years of age, and males are affected predominantly versus females with a 4.0:1 ratio [1].

Autism is a type of pervasive developmental disorder; it is a set of disorders that includes Asperger syndrome, pervasive developmental disorder "not" otherwise specified, Rett syndrome, and childhood disintegrative disorder. Together, autism, Asperger syndrome, and pervasive developmental disorder are indicated as "autistic spectrum disorder" (ASD). Rett syndrome and childhood disintegrative disorder are not within the spectrum. Autism is identified by qualitative impairments in communication and social interaction and also presents with repetitive and stereotyped behavior [2].

Before age three, most present with abnormal development. Developmental regression is seen in a quarter of these children, with specific and previously acquired skills getting lost. Of adults with autism, only 15% live an independent life without support. Based on twin and family studies, several cases suggest that autism occurs due to a combination of genetic factors. It is not a result of perinatal factors or MMR vaccines [2].

Currently, there are no medications approved to treat the core symptoms of autism spectrum disorder; these core symptoms are social communication difficulties and repetitive behavior [3]. However, atypical antipsychotic medications such as risperidone showed positive outcomes when used to treat the core symptoms [4]. Also, risperidone was shown to be effective in treating behavioral symptoms such as irritability in children in placebo-controlled studies [5]. It is also shown to be safe for young autistic children [6].

Atypical antipsychotic medications such as risperidone work by blocking postsynaptic dopamine and serotonin receptors [7]. This class of medications is more advantageous in the treatment of adult psychiatric disorders and shows potential benefits in treating children with autism. Also, controlled trials are currently being conducted for the treatment of atypical antipsychotics, especially risperidone, for the treatment of adults with autism [7]. Risperidone is a potent serotonin receptor blocker with additional dopamine-blocking properties. Elevated blood serotonin concentration levels have been seen in both children and adults with autistic disorder, and clinical studies have been shown to improve autistic symptoms in the population [8].

Risperidone is tolerated among children with autism, and recent controlled trials have shown that adults with autism spectrum disorder respond to risperidone. However, what needs to be further explored is the effectiveness of risperidone in treating autism spectrum disorder when used over an extensive period and how it is tolerated. Another area to explore is how effective risperidone is in adults and whether or not cognition and other mental and neurological functions are affected.

In this systematic review, the article will explore the effects of risperidone on cognition in people with autism spectrum disorder. Also, this article will examine the safety, efficacy, acceptability, and tolerability of risperidone on core and non-core behavioral symptoms of autism spectrum disorder.

## Review

Methodology

This systematic review used the Preferred Reporting Items for Systemic Review and Meta-Analysis (PRISMA) 2020 guidelines [9].

Search Sources and Strategy

The following databases were searched for relevant literature: PubMed, PubMed Central (PMC), Medline, Cochrane Library, ResearchGate, ScienceDirect, and Zendy. Various combinations of autism spectrum disorder, risperidone, and cognition were used in all databases to search. Additional strategies were used in PubMed to search for suitable literature in PubMed’s MeSH database (("Autism Spectrum Disorder/drug therapy" [Majir] OR "Autism Spectrum Disorder/therapy" [Majir])) AND (("Risperidone/adverse effects" [Majir] OR "Risperidone/toxicity [Majir])). PubMed’s Advanced Search database was also used to search for suitable literature as follows: PubMed Advanced Search database, ((((Autism Spectrum Disorder [Text Word]) AND (Risperidone [Title/Abstract])) AND (("2013"[Date -Publication]: "3000"[Date - Publication]))) AND (Autism Spectrum Disorder)) AND (Risperidone). Table 1 illustrates the databases used and the number of papers identified for each database.

**Table 1:** Keywords/Strategy used and the number of papers identified. MeSH: Medical Subject Headings.

Search Strategy	Database Used	Number of Papers Identified
Autism Spectrum Disorder AND Risperidone	PubMed	87
Autism Spectrum Disorder AND Risperidone AND Cognition	PubMed	37
((((Autism Spectrum Disorder[Text Word]) AND (Risperidone[Title/Abstract])) AND (("2013"[Date - Publication] : "3000"[Date - Publication]))) AND (Autism Spectrum Disorder)) AND (Risperidone)	PubMed (Advance Search)	23
(( “Autism Spectrum Disorder /drug therapy” [Majr] OR “ Autism Spectrum Disorder/therapy” [Majr])) AND (( “ Risperidone /adverse effects” [Majr] OR “ Risperidone/toxicity” [Majr] ))	PubMed (MeSH)	8
Risperidone AND Cognitive Function	Pub Med	47
Autism Spectrum Disorder AND Risperidone	Cochrane Library	28
Autism Spectrum Disorder AND Risperidone AND Cognitive Function	Science Direct	62
Autism Spectrum Disorder AND Risperidone	Science Direct	132
Autism Spectrum Disorder AND Risperidone AND Cognition	Research Gate	85
Autism Spectrum Disorder AND Risperidone	Zendy	216
Total number of research papers identified		725
Number of articles after removing duplicates		492

Inclusion and Exclusion Criteria

Papers written and published in the English language were chosen, and they focused on risperidone, autism spectrum disorder, and cognition. Articles used have no specific limit on the year of publication or age group. Mixed literature was selected, and all were required to have full text available. Also, only articles with human studies were used.

Additionally, literature that focused on autistic individuals who did not take risperidone was excluded. Grey literature was also excluded, and an article with only an abstract was not used.

Selection Process

The shortlisted articles were transferred to Endnote, where duplicates were removed. Then, the articles were screened by title and abstract, which were independently accessed by the first and second authors (JH and OF). All concerns regarding eligibility conflicts were discussed among co-authors, and a mutual consensus was reached. Next, these articles were further evaluated by screening for full-text access and selecting the relevant articles. Finally, the inclusion and exclusion criteria were conducted, and articles meeting the criteria were further shortlisted.

Quality Assessment of the Study

The reduced number of articles underwent quality checks using the appropriate quality appraisal tools. Co-authors participated in the quality check process. Clinical trials were assessed using the Cochrane bias assessment tool; systematic reviews were assessed using the Assessment of Multiple Systematic Review (AMSTAR).

Data Collection Process

The articles that will be used for the systematic review were finalized, and the primary outcome was evaluated. Also, other relevant information was evaluated. The first and second authors extracted the necessary data, and the other authors were involved in finalizing the data retrieved using a data extraction questionnaire. The data extraction questionnaire was used to identify the study type, the participants and their age, and the appropriate intervention and outcome of the study.

Results

Study Identification and Selection

**Figure 1:**
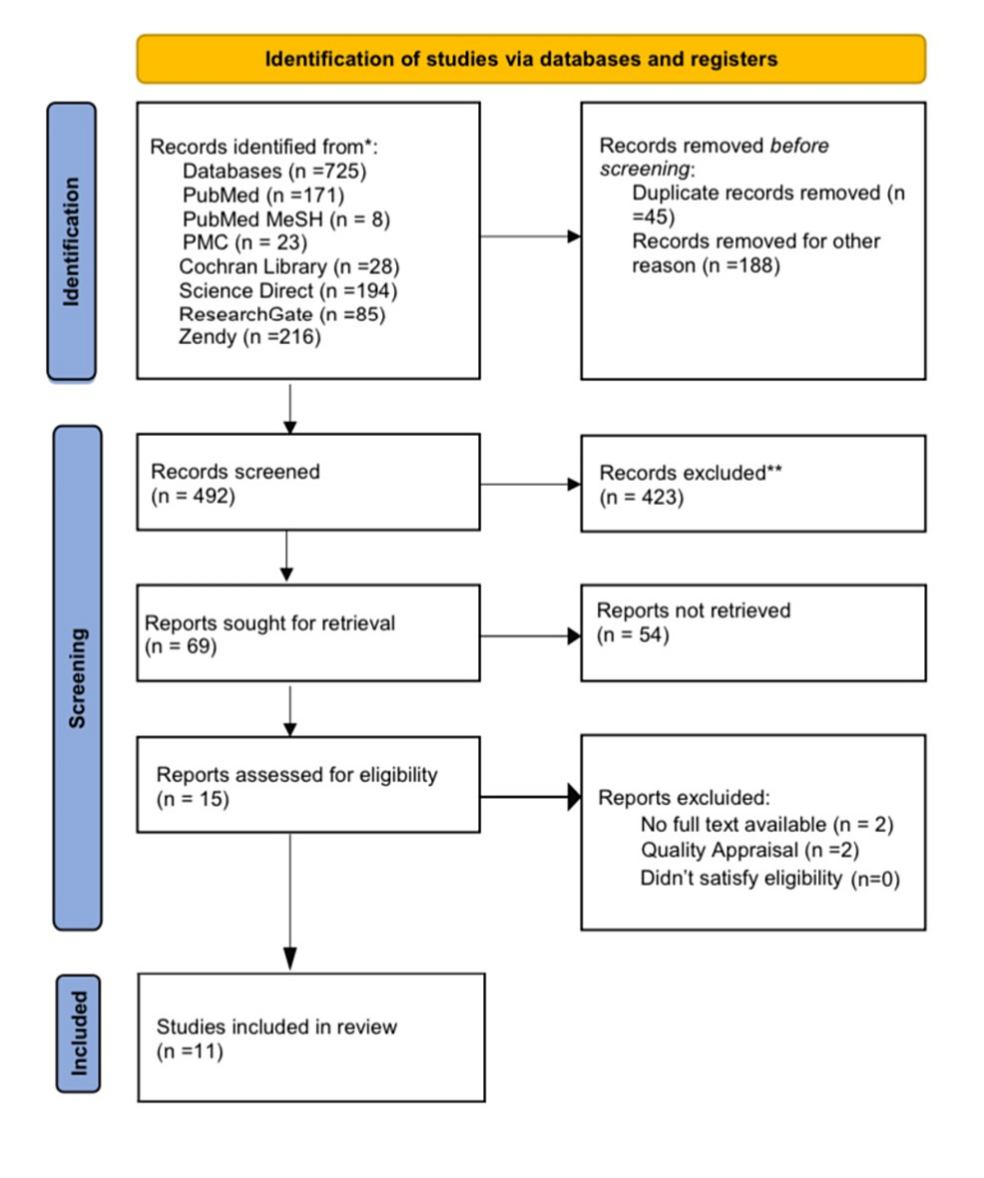
PRISMA flowchart illustrating the article selection process. PRISMA: Preferred Reporting Items for Systematic Review and Meta-Analysis; MeSH: Medical Subject Headings; PMC: PubMed Central.

There were a total of 725 relevant articles identified in the databases used; 45 duplicates were removed, and 188 were removed for other reasons. The screening was conducted by filtering through titles and abstracts and searching for full text. There were 15 articles remaining, which were evaluated for eligibility and quality. Out of these articles, 11 were chosen for review. Figure 1 illustrates the PRISMA flow chart of the selection process.

The articles were evaluated for eligibility by using the appropriate quality appraisal tools.

Table 2 illustrates the outcome of the quality appraisal.

**Table 2:** Quality appraisal using the Cochrane bias assessment tool. (+) Low risk of bias; (-) High risk of bias; (?) Unclear risk of bias.

Authors and Year of Publication	Random Sequence Generation	Allocation Concealment	Blinding of Participant and Personnel	Blinding of Outcome Assessment	Incomplete Outcome Data	Selective Reporting	Other
Aman et al. 2008	++++	++++	++++	++++	++++	++++	++++
Kent et al. 2013	++++	++++	++++	++++	++++	++++	++++
Mc Dougle et al. 2005	++++	++++	++++	++++	++++	++++	++++
McCracken et al. 2002	++++	++++	++++	++++	++++	++++	++++
Research et al. 2005	++++	++++	++++	++++	++++	++++	++++
Scahill et al. 2013	++++	++++	++++	++++	++++	++++	++++
Farmer et al. 2017	++++	++++	++++	++++	++++	++++	++++

Outcome Measured

The primary outcome identified from the finalized research papers is for autistic individuals who are taking risperidone and how safe, efficacious, acceptable, and tolerable the drug is for core and non-core behavioral symptoms of autism spectrum disorder. The secondary outcome evaluated the effects of risperidone on cognition in people with autism spectrum disorder. A few articles evaluated the long-term and short-term effects of using risperidone.

*Study Characteristics * 

There were 11 research papers reviewed with roughly 1338 participants. From the finalized studies, four were systematic reviews and meta-analyses [2,4,10,13], and seven were clinical trials [7,11,12, 14-17]. Most studies involved children and adolescents, and very few adult studies were conducted. The short- and long-term treatment of risperidone was evaluated, with dose modifications considered. Side effects were monitored to determine the safety, efficacy, acceptability, and tolerability of risperidone. Also, both the core and non-core behavioral outcomes were assessed from several study subscales in individuals with autism spectrum disorder who were treated with risperidone. Finally, cognition was also analyzed in participants with autism spectrum disorder. Table 3 illustrates the summary and characteristics of all the included studies. 

**Table 3:** Summary of the included studies. RCT: Randomized clinical trial; RUPP: Research unit on pediatric psychopharmacology; ABC: Aberrant behavioral checklist; CARS: Childhood autism rating scale; CPT: Conner’s continuous performance test.

Authors and year of publication	Type of the study	Purpose of the study	Number of participant	Results	Conclusion
Jesner et al. 2007 [4]	Systematic Review	To determine the efficacy and safety of risperidone for people with autism spectrum disorder	Three randomized controlled trials were conducted with 31-101 participants adults and children	Risperidone shows benefits in some behavioral and core symptoms. It presents with few adverse effects the most notable being weight gain	Studies show promising results in some behavioral and core symptoms of autism spectrum disorder. Long- term efficacy and safety should be further explored
Maneeton et al. 2018 [10]	Systematic Review	To determine the efficacy, acceptability and tolerability of risperidone in the treatment of children and adolescents with autism spectrum disorder	Seven randomized controlled trials were conducted with 372 randomized participants adolescents and children	The pool mean change score and the response rate for the risperidone- treated group had a greater significance than placebo - treated group in both acute and long-term treatment for both ABC-I and CARS. The discontinuation phase shows the opposite result	Risperidone is efficacious and well-tolerated in treating symptoms of autism in children and adolescents
Aman et al. 2008 [11]	RCT	To explore the effects of risperidone on cognitive process in children with autism and irritable behavior	Thirty-eight children age five to seventeen years. Twenty-nine boys and nine girls	The cognitive performance showed no decline with risperidone. Cancellation task verbal and learning tasks were superior on risperidone than on placebo. No notable difference occurred between the treatment condition of the hand-eye coordination task and the time math test task	Children with autism who were given risperidone for up to eight weeks at up to 3.5 mg dose had no harmful effect on cognitive performance
Kent et al. 2013 [12]	Clinical Trial	To evaluate the long-term safety and efficacy of risperidone in treating irritability and related behaviors in children and adolescents with autistic disorder	Seventy- nine enrolled participants ages five to seventeen years children and adolescents	The most frequent cause for discontinuation of risperidone was sparse response and adverse effects. All groups indicated improvement in the efficacy scale score during the open-label extension study	The safety profile of treatment with risperidone at optimal weight-based dose was similar to the findings observed in the double-blinded phase. Also patient encountered added improvement in irritability and other behavioral symptoms
Parr 2010 [2]	Systematic Review	To improve social function, communication, and cognitive ability and reduce the repetitive, obsessional and comorbid behaviors seen in autism	Several study designs were conducted and the studies included had at least 20 participants	Risperidone shows a more effective outcome compared to placebo for improving behavioral symptoms such as irritability, social withdrawal, stereotypy, hyperactivity and inappropriate speech at eight weeks in autistic children. Common adverse effects were identified.	Risperidone is beneficial for treating behavioral symptoms in individuals with autism. But due to adverse effects, the use in children is limited
Mano-Sousa et al. 2021 [13]	Systematic Review	To determine the efficacy of risperidone on autism spectrum disorder assessed by the Aberrant Behavioral Checklist scale	The study analyzed 2459 articles and 41 studies were meta-analysis. The population consisted of children ages two to seventeen years	There were two study groups. Short term (up to eight weeks) and long-term (after eight weeks). Risperidone proved to be effective at improving the total clinical condition of the Aberrant Behavioral Checklist scale for individuals with autism in both study group	Risperidone has shown to improve all five domains of the Aberrant Behavioral Checklist scale along with lethargy and speech. Therefore improving the overall quality of life for autistic individuals
Research Units on Pediatrics Psychopharmacology 2005 [14]	Clinical Trial	To determine the efficacy and safety of longer- term treatment with risperidone	There are 95 participants, children age five to seventeen years in a two- phase study. Phase I: 63 children Phase II: 32 children	Phase I: change in the Aberrant Behavioral Checklist Irritability scale was minute and clinically insignificant Phase II: the relapse rate was more for placebo (62%) compared to continued risperidone use (12.5%) which was statistically significant	Risperidone showed to be efficacious and tolerable for intermediate- length treatment in autistic children with specific behavioral symptoms
McDougle et al. 2005 [15]	Clinical Trial	To determine whether risperidone improves the core symptoms of autism, social and communication impairment and repetitive and stereotyped behavior	There were 101 children and adolescents age five to seventeen years (mean age 8.8 years and standard deviation 2.7) 82% were males	Treatment with risperidone for eight weeks showed no significant difference from placebo regarding qualitative impairment in social interaction and communication. However, there was an improvement in repetitive and stereotyped patterns of behavior.	Significant improvement with risperidone treatment was noted for restricted, repetitive and stereotype patterns of behavior, interest and activities in children with autism. But no significant change was noted in a deficit of social interaction and communication.
McCracken et al. 2002 [7]	RCT	To determine the safety and efficacy of atypical antipsychotic agents in children	There were 101 children. 82 boys and 19 girls.	Risperidone treatment for eight weeks at (0.5 to 3.5 mg/day) range showed a 56.9% reduction in the Aberrant Behavioral Checklist Irritable subscale versus the placebo group which showed a 14.1 percent reduction.	In the risperidone treatment group, no serious adverse effect was noted and no child withdrew from the study because of adverse events. The adverse event experienced was mild and self-limited.
Scahill et al. 2013 [16]	RCT	To examine the Aberrant Behavioral Checklist social withdrawal subscale as an outcome for social disability in children with autism spectrum disorder	There were 225 participants 187 boys and 38 girls	RUPP 1 study: 52 children on placebo and 49 children on risperidone RUPP 2 study: 49 children on risperidone and 75 children on risperidone plus parent training. RUPP2 study had a higher score on the Aberrant Behavioral Checklist which suggested that RUPP 2 study participants were less impaired. Treatment lasted for eight weeks and a decline in social withdrawal subscale score was noted in all four groups.	The social withdrawal subscale can be used to determine the endpoint of acute treatment trials that centers on social disability in autistic children.
Farmer et al. 2017 [17]	RCT	To evaluate the effect of atypical antipsychotics on attention and short-term memory	There were 165 children ages six to twelve years	The Conner’s continuous performance test (CPT-II) and the digital span memory performance showed improvement at three weeks. No difference was observed at nine weeks.	There were no harmful results on attention and short-term memory related to the short-term use of risperidone.

Discussion

Etiology of Autism Spectrum Disorder

Twins and family studies have shown evidence that indicates that several cases of autism occur due to a mixture of genetic factors [2]. Studies done within families that have a sibling with autism show that the recurrent rate of autism in children is 2.2%, and the recurrent rate among siblings for all other pervasive developmental disorders is 5% to 6%. These findings are more than what is expected in the general population. Studies comparing monozygotic twins indicate 60% to 91% concordance for autism, which led to the conclusion that the majority of cases emerged from numerous susceptible genes and impacts from environmental and other factors. A few cases of autism can be attributed to genetic disorders such as chromosomal abnormalities, fragile X syndrome, tuberous sclerosis, neurofibromatosis type 1, and several other medical conditions. From prior research, there were no findings indicating that autism is caused by MMR vaccines or thimerosal (mercury) in vaccines. Continuous research to determine the relationship between neurophysiology, neuroanatomy, neurochemistry, and genetic factors will likely increase our perspective and unravel the intricate etiology of autism spectrum disorder [2].

History of Risperidone Usage

Risperidone was initially developed to treat schizophrenia. Since 2006, the Food and Drug Administration (FDA) has approved risperidone to treat irritability related to autism spectrum disorder [13]. The Aberrant Behavioral Checklist (ABC) Irritability subscale measured non-core behavioral features such as tantrums, aggression, and self-injurious behavior, which are known to impede the activity of daily living [16]. The approval of the Food and Drug Administration (FDA) for the treatment of core symptoms of autism spectrum disorder, like social disability, will require concrete evidence for measuring the central component of autism spectrum disorder [16].

Mechanism of Action of Risperidone

**Figure 2:**
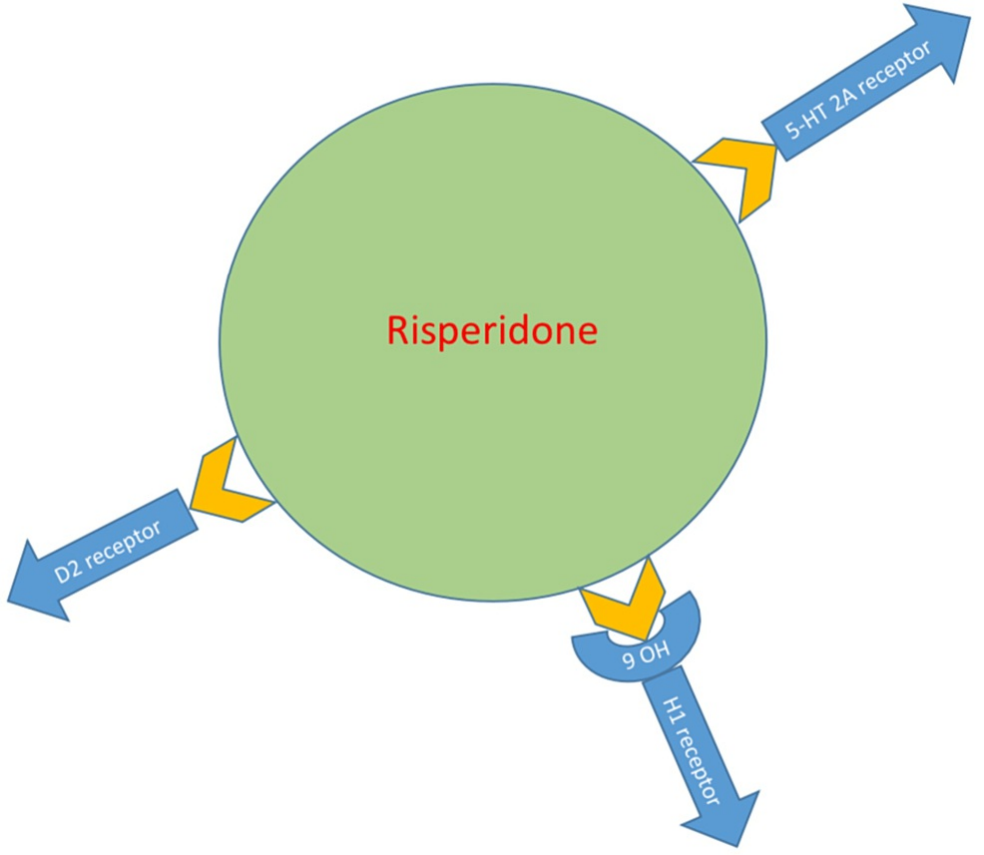
Mechanism of action of risperidone D2 receptor: Dopamine receptor; 5HT 2A receptor: Serotonin receptor; H1 receptor: Histaminergic receptor; 9OH: Paliperidone 9-OH. Figure created by the first author (JH).

Risperidone’s mechanism of action is the blockade of serotonin 2A and dopamine D2 receptors [10]. Paliperidone 9-OH-risperidone is the active metabolite in risperidone that facilitates the binding of H1 histaminergic receptors [13]. The antagonistic effect of dopamine and serotonin is thought to be the reason for the beneficial outcome of autism spectrum disorder and the reduction of extrapyramidal symptoms. The effect risperidone has on aggression, stereotypes, irritability, restlessness, and tantrums is attributed to dopamine antagonism, while the effect on restricted activity patterns, communication skills, social and emotional skills such as detachment, making eye contact, inattention, and relationships is attributed to serotonin antagonism [13]. Figure 2 illustrates the process of risperidone action.

To Determine the Safety, Efficacy, Acceptability and Tolerability of Risperidone

First, the safety, efficacy, acceptability, and tolerability have been assessed in several clinical trials for autism spectrum disorder symptoms [10]. Risperidone has shown a 70% response rate in efficacy for treating autism spectrum disorder symptoms in the short-term, long-term, and withdrawal phases in children and adolescents. In one study, acceptability, which is defined as the overall discontinuation rate, showed that risperidone treatment was comparable to placebo, and no difference in treatment was found between risperidone and placebo for tolerability [10].

Since antipsychotic drugs are often used long-term to treat autistic patients, it is important to determine the lowest efficacious dose from a safety standpoint [12]. A 26-week open-label extension study that was followed by a six-week double-blinded controlled trial phase evaluated the efficacy and safety of risperidone at a fixed weight dose range (low dose = 0.125 or 0.175 mg/day and high dose = 1.25 or 1.75 mg/day) in comparison to placebo in individuals with autism. The most common effect seen in this study was increased appetite and weight gain, which was similar to other previous studies and was not considered as severe. However, this posed a bias in severity since those who did not tolerate the risperidone in the double-blinded study were not able to continue the open-label extension study. Nevertheless, the lowest effective dose of risperidone should be used when treating individuals with autism to reduce the adverse effect of weight gain, which is dose-related [12].

In another study, both short-term use (up to eight weeks) and long-term use (after eight weeks) of risperidone in treating autistic children illustrated overall improvement of the aberrant behavioral scale in a systematic review and meta-analysis [13]. Both short-term and long-term use of risperidone significantly decreased hyperactivity, inappropriate speech, stereotype behavior, and lethargy compared to placebo. However, short-term treatment reduced inappropriate speech and lethargy more effectively. The most significant outcome for both long-term and short-term treatment was irritability and hyperactivity. For weight gain, no significant difference was noted between short- and long-term use of risperidone and placebo in the treatment of autism spectrum disorder [13].

Therefore, based on the study observation of treating all domains in the aberrant behavioral checklist scale, risperidone has the potential to treat autism spectrum disorder. This finding is promising for clinicians, especially for the treatment of speech, which in the long term will improve social interaction in children with autism spectrum disorder [13].

Also, a study of longer duration was conducted to determine the benefits involved with longer-term treatment of risperidone in children with autism spectrum disorder [14]. The study consisted of two parts and used autistic children ages 5 to 17 with severe aggression, tantrums, and self-injurious behavior who favorably responded to risperidone in a previous eight-week study. Part I of the study was a 16-week open-label risperidone treatment started at an optimal dose of 1.96 mg/day; part II of the study was a randomized double-blinded placebo substitution study for risperidone withdrawal. As with previous studies, the measured scales were the Aberrant Behavioral Checklist irritability subscale and the Clinical Global Impression Improvement scale. The results of this study illustrated that treatment with risperidone was efficacious and safe. In Part I of the study, of the 63 participants, only five were lost due to efficacy and one due to adverse effects (not weight gain). The change observed on the Aberrant Behavioral Checklist Irritable scale, and the Clinical Global Impression Improvement scale was minimal and lacked clinical significance. Over the six months, subjects had a 5.1 kg weight gain; this amount was more than expected for developmental norms. The finding could be due to a 6% dose increase in risperidone over six months. In Part II of the study, the relapse treatment rate was lower for risperidone (12.5%) compared to placebo (62.5%). The study concluded that when used for an intermediate duration of up to six months, risperidone showed consistent efficacy and favorable tolerability in autistic children with severe behavioral issues, as measured by the Aberrant Behavioral Scale and the Clinical Global Impression Improvement Scale. Since the discontinuation of risperidone resulted in the return of behavioral symptoms, it is therefore important to consider the length of continued risperidone treatment in autistic children. Furthermore, a measure of dose reduction versus complete discontinuation should be further explored [14].

Previously, the above study explored the treatment of risperidone in autistic children and adolescents with severe behavioral symptoms such as aggression, tantrums, and self-injurious behavior. Now, a similar study will be explored to determine whether risperidone treatment can improve the core symptoms in children and adolescents with autism spectrum disorder [15].

In this study, 101 children and adolescents aged 5 to 17 diagnosed with autism spectrum disorder with impaired behavioral symptoms of severe aggression, tantrums, and self-injurious behavior participated in an eight-week randomized controlled clinical trial with risperidone and placebo [15]. Similar to the previous findings, risperidone showed greater improvement than placebo on the Aberrant Behavioral Checklist scale and the Clinical Global Impression Improvement Scale. Those who responded to risperidone in the double-blinded study and the open-label study progressed to a 16-week continuation phase study. The Ritvo-Freeman Real Life Rating Scale, the Children’s Yale-Brown Obsessive-Compulsive Scale, and the maladaptive behavioral domain of the Vineland Adaptive Behavior Scale assessed core symptoms resulting from risperidone treatment [15].

Likewise, risperidone showed more efficacy than placebo for score improvement on the subscale for motor behavior, sensory response, and affective reaction on the Ritvo-Freeman Real Life Rating Scale [15]. However, there was no significant difference between risperidone and placebo for improving impaired social relatedness and language use. Similar findings were observed in a double-blinded controlled study in adults with autism and pervasive developmental disorders not otherwise specified. The above study indicated an almost significant difference between placebo and risperidone in the social domain of the Ritvo-Freeman Real Life Rating Scale and Aberrant Behavioral Checklist. From the result, it can be inferred that the reduction of behavioral symptoms such as tantrums, aggression, and self-injurious behavior promotes more flexibility for more social interaction. Additionally, the compulsion scale, which was determined by the modified Children’s Yale-Brown Obsessive Compulsive Scale, showed a greater reduction in repetitive behavior for risperidone compared to placebo. Besides, risperidone showed more efficacy than placebo in reducing the overall maladaptive behavior domain on the Vineland Adaptive Behavior Scale [15].

Therefore, treatment with risperidone for eight weeks showed no significant difference from placebo in communication and social interaction; however, improvement was observed in repetitive and stereotyped behavior patterns, activities, and interests. This pattern of response continued with an extra 16 weeks of risperidone treatment [15].

McCracken et al. further explored the clinical trial from the Autism Network of the Research Units on Pediatric Psychopharmacology [7]. Children and adolescents aged 5 to 17 weighing a minimum of 15 kg with autism and behavioral symptoms of tantrums, aggression, and self-injurious behavior were selected. Also, risperidone was shown to improve the stereotype and hyperactive behavior subscales on the Aberrant Behavioral Checklist, with no change observed in the score for social withdrawal and inappropriate speech. The adverse effect that showed a significant difference between risperidone and placebo was weight gain, and the increase was mild to moderate [7].

McCracken et al. concluded that risperidone was demonstrated to be safe and efficacious in the short-term treatment of autism in children and adolescents with aggression, tantrums, and self-injurious behavior. Additionally, risperidone demonstrated favorable outcomes compared to placebo for treating stereotypic behavior and hyperactivity. The adverse effect that demonstrated a significant difference was weight gain [7]. 

Subsequently, Scahill et al. explored the Aberrant Behavioral Checklist Social Withdrawal subscale to determine the result for social disability in autistic children using data from two randomized clinical trials. In trial one (RUPP 1), autistic children aged 5 to 17 were randomized to risperidone (N=49) or placebo (N=52) in a double-blinded study. In trial two (RUPP 2), autistic children aged 4 to 14 were assigned randomly to open-label risperidone only (N=49) or risperidone and parent training (N=75) [16].

This trial was conducted for eight weeks, and the measures used were the Vineland Adaptive Behavioral Scale, Aberrant Behavior Checklist, Clinical Global Impression Scale, and Child Symptom Inventory [16]. After the eight-week treatment ended, a decline was seen in all categories on the Social Withdrawal subscale, which resulted in treatment improvement with risperidone compared to placebo. The categories were (placebo in RUPP 1), (double-blinded risperidone in RUPP 2), (risperidone only in RUPP 2), and (risperidone plus parent training in RUPP 2). The findings from this study indicated the need for continuous clinical trials focusing on social interaction, repetitive behavior, and communication in all domains [16].

The Effect of Risperidone on Cognitive Process in Autism Spectrum Disorder

For children and adolescents who are taking atypical antipsychotics, the cognitive effects of this class of drug have not been widely explored, and most studies are conducted with adults with other psychiatric illnesses such as schizophrenia. A common side effect of antipsychotics is sedation, and it has been speculated that sedation and/or cognitive blunting, along with early treatment, may worsen cognition. However, autistic children treated with risperidone reported that sedation usually disappears after two to four weeks of the last adjusted dose. This suggests that cognitive impairment risk is greatest in the first weeks of treatment [11].

Moreover, understanding of the cognitive effect of atypical antipsychotics such as risperidone came from adult literature on the treatment of schizophrenia. These studies showed progress in attention, visuospatial processes, and executive function, and in other studies, positive results were seen in verbal learning, verbal fluency, and visuomotor tracking. There were no adult studies with atypical antipsychotics showing adverse effects on cognition [11].

Farmer et al. explored one particular study evaluating 14 children diagnosed with pervasive development disorder and irritable behavior. Children who changed treatment from risperidone to placebo experienced notable regression in attention while, improved performance was observed in those who remained on risperidone [17].

Similarly, in one study, 24 children were tested who had autism spectrum disorder (autism, pervasive developmental disorder, not otherwise specified, and Asperger‘s disorder). Fifty percent of these children were tested on attention (focused and divided attention tasks) for 24 weeks; half of the participants were withdrawn from risperidone and given the placebo treatment. The outcome showed a reduction in performance on divided attention tasks for children taking a placebo compared to those continuing on risperidone [11].

In addition, cognitive functions were assessed in 38 autistic children on risperidone who met the cognitive measure requirement; these participants’ IQs were higher, and their ages were more advanced than in previous studies [11]. Results indicated that there was no significant decline in cognition in areas of measured attention (Cancellation task, timed math test), hand-eye coordination (Purdue Pegboard), and short-term verbal memory (verbal learning task). There were notable improvements with risperidone observed in some areas of cognition, such as the cancellation task (correct detection) and the verbal learning task (correct recognition). Equal improvement was observed in the spatial memory task (difference score). It was also observed that autistic children using risperidone showed favorable outcomes versus placebo on verbal learning, such as recognition performance, but no improvement was observed in short-term recall or delayed recall. There was equal improvement observed in delayed spatial memory for children taking risperidone versus placebo. It is extremely difficult to assess children with autism and associated irritable behavior for cognitive change. Therefore, the data collected from this study gave important details on cognitive-motor tasks for this population group. The easiest cognitive task for these children to understand was the Purdue Peg Board Task [11]. Table 4 illustrates the results of the cognitive process in individuals using risperidone.

**Table 4:** Cognitive process outcome in individuals using respiridone.

Individuals	Cognitive Process	Outcome
Adults	Attention	Improve/Increase
Adults	Visuospatial Process	Improve/Increase
Adults	Executive Function	Improve/Increase
Adults	Verbal Learning	Improve/Increase
Adults	Verbal Fluency	Improve/Increase
Adults	Visuomotor Tracking	Improve/Increase
Children/Adolescents	Attention (focused and divided)	Improve/Increase
Children/Adolescents	Measured Attention (time math test)	No improvement/Remain
Children/Adolescents	Hand-eye coordinator (Purdue pegboard)	No Improvement/Remain
Children/Adolescents	Cancellation Task (correct detection)	Improve/Increase
Children/Adolescents	Verbal Learning Test (correct recognition)	Improve/Increase
Children/Adolescents	Spatial Memory Task (Dot Test, difference score)	Improve/Increase
Children/Adolescents	Verbal Learning Test (short-term recall)	No Improvement/Remain
Children/Adolescents	Verbal Learning Test (delayed recall)	No Improvement//Remain
Children/Adolescents	Delayed Spatial Memory	Improve/Increase

Even though this study shows promising cognitive responses in children with autism taking risperidone, it should be kept in mind that this is an exploratory study with a small sample size due to the difficulty of testing cognition in this population. Also, it is noteworthy to mention that the sample size evaluated had a higher IQ and was older, which could have altered the outcome of the findings. Nevertheless, this study did not indicate risperidone’s contribution to functional deficits in autistic children, and there were indications of improvement in some variables [11].

Limitations

Most of the randomized control trials in this study are of short duration, which poses a challenge in assessing risperidone efficacy and side effects with long-term treatment. Second, some of our studies did not adjust for risperidone dosage, which has left doubt about the lowest effective dosage needed. Also, the studies had a small sample size and most involved children. Two of the rating scales (Ritvo-Freeman Real Life Rating Scale and Children’s Yale-Brown Obsessive Scale) used to evaluate core symptom improvement with risperidone treatment were modified, and therefore, the reliability and validity of these scales should be interpreted with caution. Finally, there were not enough studies on cognition in children and adolescents, and most of the adult studies on cognition focused on other psychiatric disorders, such as schizophrenia.

## Conclusions

This systematic review examined the effects of risperidone on cognition in people with autism spectrum disorder. The primary outcome also examined the safety, efficacy, acceptability, and tolerability of risperidone on core and non-core behavioral symptoms of autism spectrum disorder. Autism spectrum disorder comprises a group of disorders that includes autism, Asperger syndrome, and pervasive developmental disorder "not" otherwise specified. The core features are qualitative impairments in communication, social interaction, and repetitive stereotyped behavior. Autism spectrum disorder is sometimes accompanied by non-core behavioral features such as aggression, tantrums, and self-injurious behavior. Studies showed improvement in core symptoms of communication, social interaction, and repetitive stereotyped behavior, along with non-core symptoms of aggression, tantrums, and self-injurious behaviors, on several behavioral rating scales. Risperidone is well tolerated, acceptable, safe, and efficacious; the most prominent side effect observed is weight gain, which was shown to be mild and self-limiting in several studies. Most of the cognitive effects of risperidone are understood from adult studies of other psychiatric disorders, and there were no adverse effects related to cognition noted in adult studies of risperidone treatment. In autistic children and adolescents, risperidone treatment did not cause functional deficits, and there were indications of improvement in some variables. The treatment of risperidone in adults with autism spectrum disorder is less explored and requires further focused studies. In this systematic review, most of the trials consisted of small sample sizes and were of short duration. This limitation should be further explored, as it can be beneficial for clinical practice.
